# Use of Sulphate of Iron in Chancre and Gonorrhea

**Published:** 1849-12

**Authors:** 


*Use of Sulphate of Iron in Chancre and Gonorrhea.*—[An anonymous correspondent of the Lancet says :]

The whole class of caustic agents, when applied to the Hunterian chancre (though the potassa fusa cum calce be used till the ulcer be "punched out," as recommended by M. Ricord) form an eschar with pus still secreting ; in fact, the morbid cells have not been destroyed. The alkaloids and hydro-carbons are equally inefficacious.

If a chancre be perfectly freed from its eschar and the enclosed pus, at the bottom of the excavation may be observed minute white points or germs, secreting, slowly, the morbid virus. If, now, the proto-sulphate of iron, minutely pulverized, be dropped into this excavation, the parts will instantly assume a charred appearance, the metal is absorbed into the tissue, the morbid cells or germs will instantly cease to secrete pus, the cleared cavity will shortly granulate, and a smooth surface, without induration, will be the result of the use of the proto-sulphate of iron. The chancre is destroyed.

It is known to chemists, that the proto-sulphate of iron absorbs large volumes of oxygen and nitrous oxide gases. The proto-sulphate of iron, I have observed to be the most powerful agent for arresting decomposition in animal and vegetable substances. Inflammation and decomposition in the living tissue is likewise arrested by it.

In gonorrhea, we have now an agent arresting the morbid cellular action, in the salts, which should be used in solution super-saturated.

In leucorrhea, and in simple ulcers, the morbid action is arrested or peroxidized by this metallic salt.

Large doses of this salt have been exhibited in obstinate diarrhea, with great benefit.

The action of this salt will produce a great change in superseding mercury in the treatment of diseases of specific origin.—*Lancet.*

